# Quantum correlations which imply causation

**DOI:** 10.1038/srep18281

**Published:** 2015-12-17

**Authors:** Joseph F. Fitzsimons, Jonathan A. Jones, Vlatko Vedral

**Affiliations:** 1 Singapore University of Technology and Design, 20 Dover Drive, Singapore 138682.; 2 Centre for Quantum Technologies, National University of Singapore, 3 Science Drive 2, Singapore 117543; 3 Clarendon Laboratory, Department of Physics, University of Oxford, Parks Road, Oxford OX1 3PU, U.K.; 4 Department of Physics, National University of Singapore, 2 Science Drive 3, Singapore 117542.

## Abstract

In ordinary, non-relativistic, quantum physics, time enters only as a parameter and not as an observable: a state of a physical system is specified at a given time and then evolved according to the prescribed dynamics. While the state can, and usually does, extend across all space, it is only defined at one instant of time. Here we ask what would happen if we defined the notion of the quantum density matrix for multiple spatial and temporal measurements. We introduce the concept of a pseudo-density matrix (PDM) which treats space and time indiscriminately. This matrix in general fails to be positive for measurement events which do not occur simultaneously, motivating us to define a measure of causality that discriminates between spatial and temporal correlations. Important properties of this measure, such as monotonicity under local operations, are proved. Two qubit NMR experiments are presented that illustrate how a temporal pseudo-density matrix approaches a genuinely allowed density matrix as the amount of decoherence is increased between two consecutive measurements.

Ever since the pioneering work of Bell^1^,^2^, the study of quantum correlations has proved fertile ground for gaining insight into fundamental physics. Much of that progress has been focused on spatial correlations, in the form of entanglement and quantum discord^3^,^4^,^5^, but a number of authors have extended this approach into the time domain. In particular Leggett and Garg showed that quantum systems exhibit a form of temporal correlation which cannot be accounted for by any macro-realistic theory^6^. Similarly it has been shown that assumptions of realism and locality in time lead to a form of temporal Bell inequality, which again can be violated by quantum systems^7^. Here we take a different approach: assuming quantum mechanics *a priori*, and examining the correlations which can arise. This is in line with the approach taken by a number of authors in recent years who have sought to examine the role of causality in quantum systems^8^,^9^,^10^,^11^,^12^,^13^. Quantum states which violate the Leggett–Garg inequality necessarily exhibit the causal correlations we identify, and hence recent experimental demonstrations of violations of such inequalities may constitute a limited observation of such causal correlations^14^,^15^,^16^,^17^,^18^.

In non-relativistic quantum mechanics, each system in a multi-system quantum state is assigned a separate Hilbert space and these spaces are connected through the tensor product structure. The tensor product indicates that these systems are to be treated separately, though the joint state is also well defined at each instant of time. Here we explore extending this notion to different instances in time and assigning a Hilbert space to each different instant in time in much the same way as it is done in space. The resulting spatio-temporal state is then investigated.

First we introduce the standard density matrix in quantum physics for qubits, although our ideas apply to subsystems of any dimensionality. We then show how to extend the concept of the spatial density matrix to different instances in time. The difference between spatial and temporal correlations is investigated through the introduction of the causality monotone, which is meant to capture the degree of “temporalness” in any quantum correlations. Finally, we present experiments using an NMR implementation that illustrate the basic properties of the pseudo-density matrices. Our proposal to treat spatial and temporal correlations within the same quantum formalism clearly still discriminates between the two, albeit imperfectly: when the pseudo-density matrix fails to be positive, this means that it necessarily contains a temporal element; the converse of this is not true, however, as the pseudo-density can be positive without implying spacelike separation.

## Pseudo-density matrices

The density matrix can be viewed as a probability distribution over pure states, with 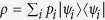, where *p*_*i*_ is the probability of a given pure state 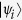 occurring. Given a density matrix *ρ*, the expectation value of a particular Pauli operator *P* is 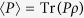. As the *n* qubit Pauli operators along with the identity form a basis for the space of Hermitian operators, and any density matrix *ρ* is necessarily Hermitian, it follows that any *ρ* can be written as 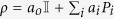, where *P*_*i*_ is the *i*th Pauli operator on *n* qubits, and 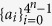 are real numbers. Further, since Pauli operators are traceless, and all density matrices have unit trace, we have *a*_0_ = 1/2^*n*^ and the expectation value for *P*_*j*_ is then given by


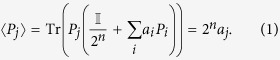


Thus we have an alternate formulation of the density matrix in terms of the expectation value of Pauli operators,


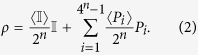


As we are interested in discussing correlations, we can express each *n* qubit Pauli operator as the product of single qubit operators, yielding


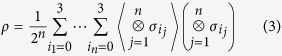


where 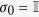, *σ*_1_ = *X*, *σ*_2_ = *Y* and *σ*_3_ = *Z*.

This above equation can be taken as defining a generalization of the density matrix. We consider a set of events 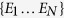, where at each event *E*_*j*_ a von Neumann measurement of a single qubit Pauli operator 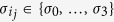 can be made. For a particular choice of Pauli operators 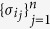, we take 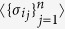 to be the expectation value of the product of the result of these measurements. Then we can define a pseudo-density matrix


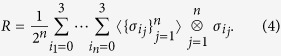


In order to compute *R* using the above equation only the expectation values for possible measurements are required. In what follows we will assume that the dynamics between measurement events are in accordance with non-relativistic quantum mechanics, and that the action of each measurement is to project onto the eigenspace of the measurement operator corresponding to the measurement result. We will assume that the underlying dynamics are non-relativistic, and so the resulting PDM will not in general be Lorentz covariant. However, we note that the general definition of the pseudo-density matrix in terms of expectation values of measurements, given above, does not assume a particular model of underlying dynamics, and thus a PDM can be reconstructed from experimental results or produced from predictions of other theories without implicitly assuming non-relativistic quantum mechanics.

In the case where the measurement events *E*_1_ … *E*_*n*_ correspond to simultaneous measurements of distinct subsystems of a quantum system, or when the measurements occur on systems which are non-interacting between measurement events, then *R* reduces to the standard *n*-qubit density matrix. In non-relativistic quantum mechanics, only simultaneous measurements are gauranteed to result in a valid density matrix, as the theory allows for low amplitude disturbances to propagate at an unbounded rate. This allows for sufficiently weak causal relationships to be established over long ranges arbitrarily quickly. However, for systems in which superluminal signalling is heavily supressed (for example for systems restricted to sufficiently weak local interactions), *R* can still be expected to approximate a conventional density matrix. This is due to the fact that if measurements are spacelike separated, then any evolution of the system between measurements must act (approximately) locally on each system, rather than allowing for interaction between systems. However, as there is no notion of separate systems inherent in the definition of *R*, it allows us to describe correlations between measurement events which are not spacelike separated, for example encapsulating the possibility of multiple measurements made at different points in time on a single system. This is a generalization of the notion of a quantum state extended across spacetime, rather than the usual restriction to some fixed time present in non-relativistic quantum mechanics. We note that other authors^11^,^19^ also considered introducing a product structure into temporal correlations, but in somewhat different contexts.

## Properties

This pseudo-density matrix inherits many of the properties of a standard density matrix. We now examine some of these properties and prove that they hold for all pseudo-density matrices.

### Hermiticity

All pseudo-density matrices are necessarily Hermitian.

*Proof:* By definition, a pseudo-density matrix is a weighted sum over Pauli matrices. As Pauli matrices are Hermitian, and all weights are real (being an expectation value divided by the dimensionality of the system), the resulting PDM is necessarily Hermitian.

### Unit trace

All pseudo-density matrices have unit trace.

*Proof:* This property again follows from the fact that PDMs are defined as a sum over Pauli matrices. Other than the identity, all such matrices are traceless. Thus, when taking the trace of a PDM, only the weight for the identity term contributes. Thus we have


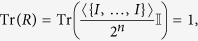


where the second equality follows from the face that the expectation value 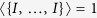 for all systems.

### Partial trace

Given a pseudo-density matrix *R*_*AB*_ defined over two sets of events *A* and *B*, the pseudo-density matrix obtained from the set of events *A* can be obtained from *R*_*AB*_ by tracing over the subsystem corresponding to *B* (i.e. *R*_*A*_ = Tr_*B*_(*R*_*AB*_)).

*Proof:* From the definition of the pseudo-density matrix, we have


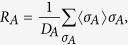


and


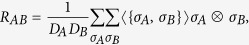


where *D*_*A*_ and *D*_*B*_ are the dimensions of the systems corresponding to *A* and *B* respectively. Thus we have


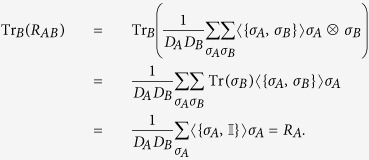


The above properties do not depend on the physics governing the underlying system. We now show that if the underlying system is governed by non-relativistic quantum mechanics, then the pseudo-density matrix captures all correlations in the system. We will index the measurement events in such a way that event *j* occurs no later than event *j* + 1. Without loss of generality, we can assume that all measurement events occur on a system initially in state *ρ*, which evolves unitarily according to *U*_*j*_ between measurement events *j* and *j* + 1. We will take the number of distinct times at which measurements occur to be *T* and take the total number of measurement events at each time *t* to be *m*_*t*_. In this case the pseudo-density matrix satisfies the following additional properties.

### Measurements

The pseudo-density matrix contains information not only about Pauli measurements, but also about the expectation value of the product of any set of local measurements with eigenvalues restricted to ±1. For a set of measurement operators {*M*_*j*_}, with eigenvalues chosen from {−1,  1}, the expectation value for the product of their outcomes is given by


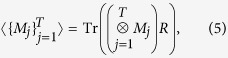


where *M*_*j*_ is defined over the set of *m*_*j*_ qubits on which measurement events occur at time *j*.

*Proof:* In order to prove this property, it is sufficient to show that expectation values for measurements of this are simply a linear combination of expectation values for Pauli measurements. This is, taking 
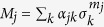
 where the superscript on 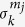 denotes that it is an *m*_*j*_-qubit Pauli operator, the expectation value is given by


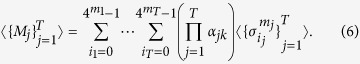


The above equation can be obtained directly from Eq. 5 by substituting in the definitions of *R* and each *M*_*j*_, and so it suffices to show that this linearity relation holds.

In general, 
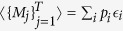
 where *p*_*i*_ is the probability of obtaining a product of measurement results equal to *ϵ*_*i*_. For non-relativistic quantum systems,


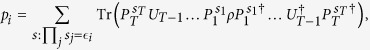


where 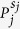 is the projector onto eigenspace of *M*_*i*_ corresponding to eigenvalue *s*_*j*_. If no constraint is placed on the spectrum of *M*_*j*_, then 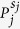 may be a high degree polynomial in *M*_*j*_. However, in the case where the eigenvalues of *M*_*j*_ are restricted to the set {−1, 1}, the projectors can be written as 
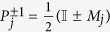
, and thus *p*_*i*_ has degree at most two in each of the measurement operators. Furthermore, since 
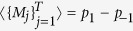
 the quadratic and constant terms cancel, leaving the purely linear expression in Eq. 6. Thus, expectation values for measurements can be computed for the pseudo-density matrix in an identical way to that used for conventional density matrices, provided that the measurement operators have only eigenvalues chosen from {−1, 1}. This shows that the pseudo-density matrix captures more that simply correlations between Pauli measurements, but rather contains the necessary information to predict measurement outcomes for a wide range of measurements, without any further knowledge of the underlying dynamics giving rise to a particular PDM other than that they are governed by non-relativistic quantum mechanics. This shows that the information captured by the PDM is invariant under local change of basis.

## Causality

While the previous section examined points of commonality between pseudo-density matrices and conventional density matrices, we now turn to a difference between the two. All density matrices are positive semi-definite matrices with unit trace, and any matrix satisfying these requirements can be interpreted as a density matrix. The main difference between a pseudo-density matrix *R* and a conventional density matrix, then, is that *R* is not necessarily positive semi-definite. To see this, we consider the case of a single physical qubit with two separate measurement events. We take the qubit to be initially in the state 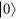 and assume that evolution between measurement events corresponds to the identity operator. In this case the expectation values are all zero, except for 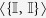, 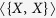, 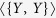, 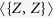, 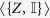, and 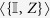, which are all equal to one. From these expectation values, we obtain a pseudo density matrix


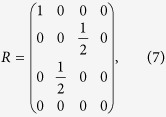


which has eigenvalues 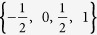. The existence of negative eigenvalues implies that *R* is not positive-semi definite.

Any *R* which is positive semi-definite can be interpreted as a conventional density matrix, for which it is possible to duplicate the correlations present with spacelike separated quantum systems. However, when *R* has negative eigenvalues, it cannot be interpreted in this way, implying that there must exist a causal relation between events, although the causal order is not specified.

## Measuring causal relationships

The causal relationship embodied in certain pseudo-density matrices has some similarities to another form of uniquely quantum correlation, entanglement, and we can define an analogous measure of causal correlations. In order for a function *f*(*R*) to be considered a causality monotone we require the following criteria to hold:

1. *f*(*R*) ≥ 0, with *f*(*R*) = 0 if *R* is completely positive, and *f*(*R*_2_) = 1 for any *R*_2_ obtained from two consecutive measurements on a single qubit closed system,
2. *f*(*R*) is invariant under local unitary operations,
3. *f*(*R*) is non-increasing under local operations, and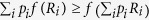.

These criteria correspond almost exactly to the criteria for an entanglement monotone^20^,^21^, except that criterion three is somewhat weakened. An entanglement monotone is required not to increase on average under local operations and classical communication, however any processing based on classical communication would constitute a causal relationship and hence is excluded.

As we have shown, any pseudo-density matrix which embodies some form of causal relationship must have at least one negative eigenvalue. Since such matrices are Hermitian (and hence have real eigenvalues) and have unit trace, it follows that the trace norm is strictly greater than unity. On the other hand, if all eigenvalues are positive, the trace norm is exactly equal to unity.

This leads us to define a measure based on the trace norm, 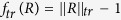. As we have seen, 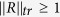 for all valid pseudo-density matrices, and hence *f*_*tr*_(*R*) ≥ 0. Further, *f*_*tr*_(*R*) = 0 trivially for all positive semi-definite *R*, and from the previous example it is clear that *f*_*tr*_(*R*_2_) = 1 for at least one choice of *R*_2_. Since the trace norm is unitarily invariant, the first and second criteria for *f*_*tr*_ to be a causality monotone are satisfied. Similarly, by applying Stinespring dilation to represent local quantum operations as unitary operations on a larger Hilbert space, the third criterion follows directly since the trace norm is non-increasing under partial trace. The final criterion follows from the triangle inequality since 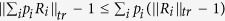 and hence 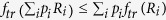. Thus *f*_*tr*_ is a causality monotone.

## Experimental determination of 2-site pseudo-density matrix

Naively it would appear that non-destructive single qubit measurements are necessary in order to perform the multi-event measurements required for tomography of a pseudo-density matrix. This would rule out the possibility of reconstructing a pseudo-density matrix in either NMR or quantum optics, two of the most established testbeds for quantum physics. Fortunately, however, it is possible to circumvent the limitations imposed by ensemble measurements by making use of an ancilla qubit to record the parity of the local Pauli measurement results. Thus it is possible to recover their product by measuring a single spin, similar to the approach advocated in^22^.

**Figure 1:**
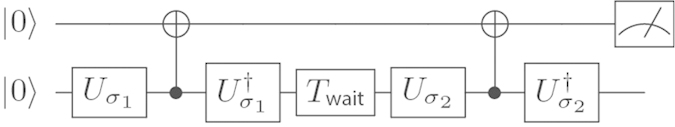
A quantum circuit for measuring correlations in time.

The simplest system for which *R* can have negative eigenvalues contains two measurement events. These can be made either on the same qubit or seperate qubits. However in order to observe both causal and acausal correlations in the current generation of experiments we focus on measurements separated by a variable time on a single qubit, as in the circuit shown in Fig. 1 which accomplishes the measurement {*σ*_1_, *σ*_2_}. In the figure, 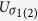 is the unitary operation mapping the ±1 eigenstate of the Pauli operator *σ*_1(2)_ onto the ±1 eigenstate of *Z*. Between measurements we allow a period of free evolution, during which the primary qubit undergoes decoherence, and we calculate a pseudo-density matrix *R*_*T*wait_ for a range of waiting times.

**Figure 2:**
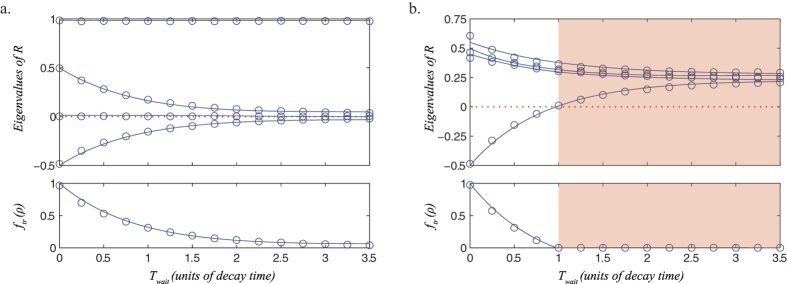
Eigenvalues of *R* and value of *f*_tr_ as a function of *T*_wait_. In (**a**) the system starts in a pseudo-pure state and undergoes dephasing noise, while in (**b**) the system starts in a mixed state and undergoes depolarising noise. The circles indicate data points obtained from experiment, while the solid lines indicate the best fit for the relevant theoretical models. These models each take 3 parameters to describe the initial state of the system and either 1 and 3 parameters, respectively, to parametrize the noise. The red region indicates the time period in which all resulting pseudo-density matrices are acausal. Error bars (not shown) are comparable to the symbol sizes.

Figure 2 shows the results of our NMR experiments, plotting the eigenvalues of the pseudo-density matrices as a function of time, along with the corresponding *f*_*tr*_, in each of two settings. Figure 2(a) shows the results of purely dephasing noise acting on an initial state pseudo-pure state 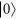. Here the pseudo-density matrix starts with a single negative eigenvalue, which tends towards zero from below as the waiting time is increased. The pseudo-density matrix never becomes positive semi-definite (and hence acausal) because the decoherence brings it towards a matrix which is rank deficient, and so the minimum eigenvalue approaches, but never quite reaches, zero.

In order to observe a sharp transition between causal and acausal pseudo-density matrices it is necessary both to start with a mixed initial state, and to allow depolarizing decoherence, which is the case considered in Fig. 2(b). Now we observe a transition between causal and acausal pseudo-density matrices as the minimum eigenvalue crosses the zero threshold, a phenomenon reminiscent of entanglement sudden death^23^.

## Methods

NMR experiments were performed on a Varian Unity Inova spectrometer with a nominal ^1^H frequency of 600 MHz using a HF{CP} probe with pulsed field gradients. The NMR sample comprised ^13^C-labelled sodium formate dissolved in D_2_O at 20 °C, providing a heteronuclear two-spin system. The ^1^H spin was used as the primary qubit and the ^13^C spin as the ancilla. Both spins were placed on resonance, so that the Hamiltonian took the form of a spin–spin *ZZ* coupling of 194.7 Hz, and the B_1_ field strengths were adjusted to give nutation rates of 12.5 kHz. The measured relaxation times were T_1_ = 7.8 s and T_2_ = 3.2 s for ^1^H and T_1_ = 16.3 s and T_2_ = 6.7 s for ^13^C. An inter-scan delay of 60 s ensured that the spin system began each experiment close to its thermal state.

Quantum logic gates were implemented using standard approaches^24^,^25^. Single qubit rotations in the *XY*-plane were implemented using BB1 composite rotations^26^,^27^, while *Z*-rotations were implemented as frame rotations^28^ which were propagated through the pulse sequence^29^ to points where they could be dropped. Pseudo-pure two-qubit states were prepared using the method of Kawamura *et al.*^30^; for pseudo-pure single qubit states the thermal state was used directly. NMR spectra were processed using home written software and the intensity of the ^13^C doublet determined by combining separate integrals for the two components; all integrals were normalised using a reference spectrum.

Instead of using natural decoherence during *T*_wait_, controllable dephasing of the primary qubit was implemented using the diffusive suppression of pulse field gradient spin echoes^31^ as described by Cory *et al.*^32^. This can be converted to controlled depolarization by using single qubit rotations to apply the dephasing around the *X*, *Y* and *Z*-axes in turn. This process also dephases the ancilla qubit, but leaves its *Z*-component unaffected as the ancilla does not experience the single qubit gates.

Theoretical predictions for the behaviour of the causality monotone were computed using a four variable model for the dephasing experiments and a six variable model for the depolarization experiments. Three variables of each model correspond to the expectation values for *X*, *Y* and *Z* for the initial state of the qubit. An exponential fall-off in correlations which anti-commute with individual error terms was assumed, consistent with a constant rate of Pauli errors. For the dephasing experiments, the rate of Pauli errors was assume to be non-zero for only *Z* errors, leading to a fourth parameter. For the dephasing experiments three parameters corresponding to constant rates of *X*, *Y* and *Z* errors were introduced. Least squares fitting was then used to fit the theoretical models obtained in this way to the value of the eigenvalues of the reconstructed PDMs obtained from experiment.

## Additional Information

**How to cite this article**: Fitzsimons, J. F. *et al.* Quantum correlations which imply causation. *Sci. Rep.* **5**, 18281; doi: 10.1038/srep18281 (2015).
